# Distal bile duct cancers complicated with cholangiobronchopleural fistula after ERCP: A case report

**DOI:** 10.3892/ol.2014.2342

**Published:** 2014-07-10

**Authors:** CHANGSONG ZHANG, GUANGSHUN YANG, YANG LING, GUIHUA CHEN, TIANBAO ZHOU

**Affiliations:** 1 Clinical Oncology Laboratory, Changzhou Tumor Hospital, Medical College of Soochow University, Changzhou, Jiangsu 213002, P.R. China; 2 The Eastern Hepatobiliary Surgery Hospital, The Second Military Medical University, Shanghai 200438, P.R. China; 3 The Hepatic Surgery Center, The Third Affiliated Hospital, Sun Yat-Sen University, Guangzhou, Guangdong 510630, P.R. China; 4 The Hepatobiliary Surgery Centre, The Affiliated Ningbo No. 2 Hospital, Ningbo University School of Medicine, Ningbo, Zhejiang 315010, P.R. China

**Keywords:** distal cholangiocarcinoma, cholangiobronchopleural fistula, endoscopic retrograde cholangiopancreatography

## Abstract

Distal (lower) bile duct cancers arise in the lower half of the biliary tree closer to the small intestine. Biliary disease complicated with cholangiobronchopleural fistula, which may occur in cases of multiple hepatobiliary stones or biliary ascariasis-associated severe infection, has rarely been reported in the literature, particularly following endoscopic retrograde cholangiopancreatography (ERCP). The present study describes the case of a 60-year-old female with distal cholangiocarcinoma complicated with cholangiobronchopleural fistula after ERCP for this rare disease. This complication was likely due to the inability to control retrograde infection following ERCP and, thus, the infection was disseminated. This resulted in mixed infection involving the diaphragm and pleura, and further penetrating the bronchus. The patient was managed with pancreatoduodenectomy and has since remained in good health.

## Introduction

Biliary disease complicated with cholangiobronchopleural fistula has rarely been reported in the literature. It may occur in cases of multiple hepatobiliary stones or biliary ascariasis-associated severe infection; however, there has been no literature in China reporting complicated cholangiobronchopleural fistula after endoscopic retrograde cholangiopancreatography (ERCP) (1,2). The present study describes a case of distal cholangiocarcinoma complicated with cholangiobronchopleural fistula in a 60-year-old female following ERCP for this rare disease. The study was approved by the ethics committee of the Eastern Hepatobiliary Surgery Hospital, The Second Military Medical University (Shanghai, China), and written informed consent was obtained from the patient.

## Case report

### Patient characteristics

**Figure 1:**
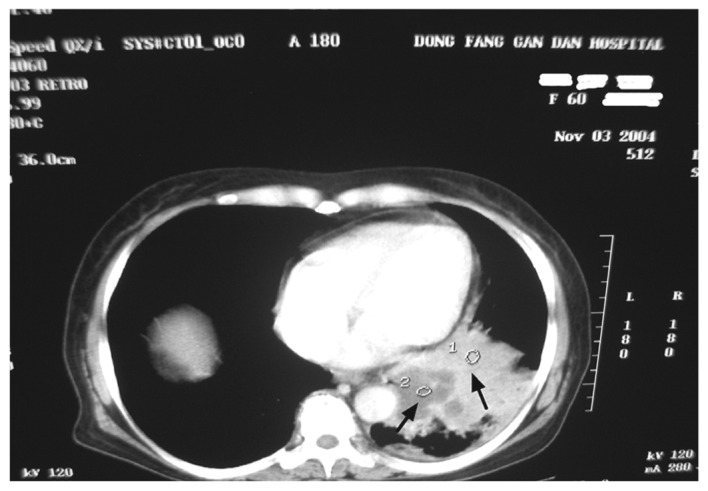
Computed tomography of the abdomen shows possible choledochoduodenal fistulas in the common bile duct (arrows 1 and 2).

The present study describes a 60-year-old female patient who was admitted to the Hepatic Surgery Center at the Eastern Hepatobiliary Surgery Hospital (Shanghai, China) on October 25, 2004 due to of icteric skin and sclera accompanied with chill and fever for more than one month. Physical examination revealed the following: Conscious; deep tenderness of the upper abdomen without rebound pain; and liver and spleen not palpable under subcostal margin. ERCP prior to admission to this hospital revealed a space-occupying lesion of the lower segment of the common bile duct associated with dilation of intra- and extrahepatic biliary ducts and cholecystitis, which were consistent with computed tomography (CT) and magnetic resonance imaging findings following admission. Laboratory evaluation revealed that the patient’s total billirubin (TBIL) and direct billirubin (DBIL) levels were 210.1 (normal range, 5.1–17.1 μmol/l) and 167.5 μmol/l (normal range, 0–6.0 μmol/l), respectively. A clinical diagnosis of carcinoma of the lower segment of the common bile duct was made. Liver protection, nutritional support and symptomatic therapies were instituted following admission. On November 1, 2004, liver function re-examination showed the following: TBIL, 29.4 μmol/l; DBIL, 18.1 μmol/l; aspartate aminotransferase, 100.6 U/l (normal range, 8–35 U/l); alkaline phosphatase, 907 U/l (normal range, 25–100 U/l); and a normal albumin level of 35.5 g/l. On November 2, 2004 (the eighth day following admission), the patient suddenly complained of chest suffocation, shortness of breath and a cough producing ~300 ml bile-like sputum per day. The patient did not experience fever, nausea or vomiting. Physical examination showed icteric skin and sclera as before; normal heart sound on auscultation; moderate coarse rale audible in the right lung; abdomen flat and soft, without tenderness or lump; shifting sound negative. An emergency CT scan was performed for the chest, both lungs and the abdomen (Fig. 1). Sputum and fistula fluid biopsy pathological findings were bile with neutrophilic leukocyte and lymphocytic infiltration. The diagnosis of a right cholangiobronchopleural fistula was made.

### Treatment

Based on the diagnosis, ultrasound-guided percutaneous transhepatic cholangiodrainage (PTCD) was instituted to eliminate jaundice, and 60 ml bile was drained promptly. The patient fasted and therapies were instituted for inhibition of bile secretion, reduction of bronchial mucous secretion, maintenance of airway passage, resolution of phlegm, protection of liver function, normalization of bile secretion, nutritional support and anti infection. The detailed protocol was as follows: i) Subcutaneous injection of 0.1 mg sandostatin three times a day on days 1 and 2 for inhibition of bile secretion; ii) ceftazidime pentahydrate, ofloxacin and metronidazo1e once a day for anti-infection effects; iii) total parenteral nutrition (TPN) support once a day; and iv) intravenous push of 60 mg ambroxol three times a day. Following this treatment, ~80 ml bile was drained by PTCD. The cough symptoms improved significantly, and the bile-like substance that the patient coughed up gradually decreased. On day 3, sandostatin (0.1 mg) was administered twice a day, and the therapies in the detailed protocol remained unchanged. Bile drainage from PTCD reduced to 20ml daily and the cough symptoms further improved, without the bile-like substance. At day 4, sandostatin (0.1 mg) was administered daily, and bile drainage from PTCD reduced to 3 ml. At day 5, sandostatin was withdrawn. The condition of the patient had become stable by the day of surgery, without cough or bile-like substance. On November 15, 2004, cholecystectomy and Roux-en-Y cholangiojejunoostomy were performed with written informed consent obtained from the patient and the patient’s family, lest the patient should not be able to tolerate pancreatoduodenectomy. Following discharge, the patient did not have any complaints or associated symptoms and, on January 25, 2005, the patient was re-admitted due to the patient’s wish for pancreatoduodenectomy. The postoperative recovery was uneventful and the patient has since remained in good health.

## Discussion

Fistula communications between the biliary tract and bronchopleural space are rare, but have been reported by Dasmahapatra *et al* (3) in advanced breast carcinoma. The most common cause of acquired pleurobiliary and bronchobiliary fistula is thoracoabdominal trauma. However, ERCP could be an incentive for cholangiobronchopleural fistula, due to its invasive means of examination and treatment, as observed in the current case. According to our analysis, the present complication was likely due to the inability to control retrograde infection following ERCP. This resulted in dissemination of the infection, causing mixed infection involving the diaphragm and pleura, and further penetrating the bronchus. As ERCP is an invasive means of examination and treatment, ERCP-associated morbidity is almost unavoidable. For example, the occurrence of hyperpancreatoamylasemia including acute pancreatitis (AP) after ERCP is as high as 40–50% (4). The most common diagnostic ERCP-associated complication is AP, and the next is cholangitis. Hemorrhage and perforation are relatively rare (5,6). Based on our experience in the present case, we suggest that inhibition of bile secretion, PTCD drainage, starvation and TPN are of primary importance, of which subcutaneous administration of sandostatin is of vital importance.

We propose that it is possible to prevent this complication from occurring. Positive, initiative, timely and complete anti-infection therapy, nutritional support and drainage (when necessary) should be considered as early as possible before performing procedures including ERCP and surgical operation, or treating hepatobiliary stones which are liable to cause infection, or any other disease which may be free of infection for the time being but may cause potential infection (7). In the case of any sign of infection, the cause should be sought as soon as possible and dealt with immediately. However, as we only have experience of one case of hepatobiliary disease-complicated cholangiobronchopleural fistula, further study is necessary to gain more experience in dealing with such a complication.

## References

[1] Habib E, Elhadad A (2003). Digestive complications of gallstones lost during laparoscopic cholecystectomy. HPB (Oxford).

[2] Delcò F, Domenighetti G, Kauzlaric D, Donati D, Mombelli G (1994). Spontaneous biliothorax (thoracobilia) following cholecystopleural fistula presenting as an acute respiratory insufficiency. Successful removal of gallstones from the pleural space. Chest.

[3] Dasmahapatra HK, Pepper JR (1988). Bronchopleurobiliary fistula. A complication of intrahepatic biliary stent migration. Chest.

[4] Nøjgaard C, Hornum M, Elkjaer M (2009). Does glyceryl nitrate prevent post-ERCP pancreatitis? A prospective, randomized, double-blind, placebo-controlled multicenter trial. Gastrointest Endosc.

[5] Williams EJ, Hamlyn A, Logan RF, Martin D, Wilkinson ML, Lombard M (2009). Consenting patients for endoscopic retrograde cholangiopancreatography: results of a survey of 182 UK endoscopists and 2059 of their patients. Eur J Gastroenterol Hepatol.

[6] Cennamo V, Fuccio L, Repici A (2009). Timing of precut procedure does not influence success rate and complications of ERCP procedure: a prospective randomized comparative study. Gastrointest Endosc.

[7] Lin CT, Hsu KF, Yu JC (2009). Choledochoduodenal fistula caused by cholangiocarcinoma of the distal common bile duct. Endoscopy.

